# Universal versus selective screening for gestational diabetes mellitus among antenatal clinic attendees in Abakaliki: using the one-step 75 gram oral glucose tolerance test

**DOI:** 10.1186/s12884-021-04168-8

**Published:** 2021-10-29

**Authors:** Silas Alegu Nwali, Robinson Chukwudi Onoh, Ikechukwu Bo Dimejesi, Vitus Okwuchukwu Obi, Sunday Emmanuel Jombo, Oghenevwogaga Obukohwo Edenya

**Affiliations:** 1 Department Of Obstetrics And Gynecoloy (Obgyn), Alex-Ekwueme Federal University Teaching Hosital (Ae-Futh), Abakaliki, Nigeria; 2 Department Of Obgyn, Federal Medical Center (Fmc), Asaba, Nigeria; 3 Department Of Chemical Patholoy, Ae-Futh, Abakaliki, Nigeria

**Keywords:** Gestational diabetes mellitus, One-step, Oral glucose tolerance test, Selective screening, Universal screening

## Abstract

**Aim:**

To compare universal screening with selective risk factor based screening for GDM, using the one-step 75 g oral glucose tolerance test (OGTT).

**Materials and method:**

A cross-sectional, comparison between universal and selective risk factor based screening for GDM, among 400 antenatal care clients at Alex-Ekwueme Federal University Teaching Hospital Abakaliki (AE-FUTHA). All the participants had 75 g OGTT at 24–28 weeks of gestation and risk factor screening for GDM. All 400 participants formed the universal group while participants with one or more of the considered risk factors formed the selective risk factor group.

Data were analyzed using IBM SPSS version 20. Statistical comparison was done using t- test for continuous variables. Logistics regression was used to determine the level of associations of the independent predictors for hyperglycemia. Level of significance was set at *P* < 0.05.

**Results:**

The point prevalence of GDM using universal and selective screening were 11.51 and 7.93% respectively, giving a selective screening miss rate of 31.11%. The sensitivity, specificity, positive predictive value and negative predictive value were 73.58, 48.82, 19.12 and 92.51% respectively for the selective risk factor based screening compared to universal screening.

On multivariate analysis; age ≥ 35 years, weight ≥ 90 kg, history of previous GDM and hypertension were significantly related to the development of hyperglycemia.

**Conclusion:**

Selective risk factor based screening missed 31.11% of patients with GDM when compare to Universal screening with one step 75 g OGTT. Universal screening for GDM using the one step 75 g OGTT is recommended for pregnant women and more studies are needed to compare pregnancy outcomes for pregnant women diagnosed with GDM with and without risk factors.

## Introduction

Gestational diabetes mellitus (GDM) is glucose intolerance with onset or first recognition during pregnancy that is not clearly overt diabetes[1, 2]. It is a common metabolic complication in pregnancy[3, 4] with increased risk of maternal and perinatal morbidity and mortality[5]. There has been an increase in the prevalence globally. It is reported to occur in 7% of pregnancies with a range of 1 to 14%[5–7], depending on the ethnic mix of the study population and the criteria used for diagnosis. In Nigeria the prevalence of GDM ranges from 2.5 to 13.9%[5, 8–11]. However a prevalence of between 3.6–25% have been reported in other African countries. The risk of developing GDM is higher in women with previous history, previous delivery of macrosomic baby, unexplained intrauterine fetal deaths, difficult delivery (ies) and maternal obesity. Others include high blood pressure, habitual smoking, increased maternal age and parity as well as in patients whose first degree relatives have diabetes mellitus[6, 8, 12–16]. More so women with a combination of risk factors have increased chances of developing GDM. Thus, Popova et al. noted that a higher BMI, abdominal circumference and polycystic ovary syndrome predict increased GDM risk.[17] Increased plasma fasting glycaemia at the first prenatal visit was associated with increased odds of developing GDM and hyperbilirubinemia[18].

The hallmark of GDM is insulin resistance associated with hyperglycemia[6] leading to increased maternal and perinatal morbidity and mortality and long term complications. Thus, there is the need for early diagnosis and adequate management of cases to avert these complications[4, 6, 12].

Currently there is lack of international uniformity in the approach to the screening and diagnosis of GDM. Controversies include universal versus selective screening, the optimal time for screening, appropriate tests and cut off values, and whether testing should be conducted in one or two steps[6]. Screening for GDM is better done between 24 and 28 weeks since fasting glucose values are lower in the first and early second trimesters in normal pregnancies, compared to the non-pregnant state[19, 20]. The most sensitive way to screen is with the OGTT[19]. Recent evidence suggest that the same test should be used for both screening and diagnosis[2, 21, 22].

National Institute for Clinical Excellence (NICE) recommends that all women with one or more risk factors for GDM should be screened using an oral glucose tolerance test (OGTT)[23]. Universally offering OGTT was associated with increased identification of women with GDM with neonatal benefits for GDM patients[24], while selective risk factor based screening miss one-third of the women with GDM[25]. The International Federation of Gynaecology and Obstetrics (FIGO) recommends universal screening for GDM using a one-step procedure and encourages all countries and its member associations to adapt and promote strategies to ensure this[26].

The Hyperglycemia and Adverse Pregnancy Outcome (HAPO) study has helped to publish the most recent diagnostic criteria by the International Association of Diabetes in Pregnancy Study Groups (IADPSG) where the same test is used for both screening and diagnosis[21, 25]. In the IADPSG diagnostic criteria for GDM one or more of the following threshold, in the 2 h 75 g OGTT, is diagnostic: fasting plasma glucose ≥5.1 mmol/l (92 mg/dl), 1 h ≥ 10 mmol/l (180 mg/dl), and 2 h ≥ 8.5 mmol/l (153 mg/dl). While NICE recommends selective screening approach[8, 23], IADPSG, ACOG and FIGO concerned with the increasing prevalence of GDM advocate universal screening[26–28].

The aim of this study was to compare the pickup rate of GDM among the antenatal clinic attendees in AE-FUTH, Abakaliki using universal and selective screening with the one-step 75 g Oral Glucose Tolerance Test.

## Materials and method

### Study design

This was a cross-sectional, comparative study of the pickup rate of GDM with universal screening and risk factor based screening among antenatal clinic attendees at AEFUTHA, Ebonyi State, Nigeria from 1st November 2017 to 30th April 2018.

Simple Random Sampling with replacement was used in this study. Participants.

were pregnant women at less than 28 weeks of gestation. Pregnant women with pre-gestational diabetes, those at more than 28 weeks of gestation as well as those with history of allergy to glucose were excluded.

### Sample size determination

The sample size was calculated using the formula for Cross-sectional study when end point is qualitative [29].

$$\rm{Sample \, size}\;=\;\lbrack{\rm{Z}}_{1-\alpha/2}{^2\rm{P}}(1-\text{P})\rbrack/\rm{d}^2$$

Where;

Z_1-α/2_ = standard normal variate, which is 1.96 at 5% type 1 error (*p* < 0.05) and power of 80%.

P = expected proportion in population based on previous studies, which here is 19% [30].

d = absolute error or precision (taking d = 0.05).

$$\begin{aligned}\rm{Sample \, size}&=\;\lbrack1.96^2\;\rm{x}\;0.19(1-0.19)\rbrack/0.05^2\\&=\;\lbrack0.729904\;\rm{x}\;0.81\rbrack/0.0025\\&=\;236.49\;=\;236\\\end{aligned}$$

Thus the minimum sample size required is 236, plus 20% attrition (47) equals 283.

Sample size for this study is therefore **283** clients.

However, the sample size was increased to **400**, to improve the power of the study.

Those recruited had 75 g OGTT after an overnight fast of 8 to 12 h duration and due counselling on the procedure. Plasma glucose measurements were carried out using the glucose oxidase enzymatic colorimetric assay (Randox Glucose Assay; United Kingdom).

The World Health Organization (WHO) [2] diagnostic criteria for GDM were used in this study.

### Ethical considerations

Ethical clearance was obtained from the Health Research and Ethics committee of the Alex-Ekwueme Federal University Teaching Hospital Abakaliki.

### Data analysis

Data were collated, tabulated then statistically analyzed using statistical Package for Social Science (IBM SPSS) software (version 20, Chicago II, USA).

The data was summarized into tables and chart. Continuous variables were presented as mean and standard deviation (Mean ± 2SD). Categorical variables were presented as frequencies and percentages. Logistics regression was used to determine the level of association of the independent predictors of hyperglycemia. The difference between the mean of the numerical variables of women who had hyperglycemia and those who did not were assessed using the t-test. Level of significance was set at *p* < 0.05.

## Results

Four hundred participants were recruited but 391 (97.75%) of them completed the study. Thus, 391 participants were analysed for the universal group while 204 participants from among the universal group with one or more risk factors for GDM were grouped into the selective group.

Majority (281; 71.87%) of the participants were between 25 and 34 years of age, of which 128 were in the selective arm. The participants predominantly had tertiary education, 72.12% (281/391) in the universal arm and 70.59% (144/204) in the selective arm. Less than half (43.22%) were civil servants. Nearly all the participants (96.68%, 378/391) were married and 68.54% (267/391) were multiparae as showed in Table 1.

**Table 1:** Sociodemographic characteristics of participants

Sociodemographic Parameter	Universal *n* = 391 (100%)	Selective *n* = 204 (100%)
Age (Years);		
< 25	44 (11.25)	9 (4.41)
25–34	281 (71.87)	128 (62.75)
35–44	61 (15.60)	62 (30.39)
≥ 45	5 (1.28)	5 (2.45)
Occupation;		
Civil Servant	169 (43.22)	98 (48.04)
Self Employed	62 (15.86)	32 (15.69)
Trader	95 (24.30)	52 (25.49)
Student	35 (8.95)	9 (4.41)
House Wife	30 (7.67)	13 (6.37)
Tribe;		
Igbo	364 (93.09)	181 (88.73)
Hausa	5 (1.28)	5 (2.45)
Others	22 (5.43)	18 (8.82)
Marital Status;		
Single	13 (3.32)	0 (0)
Married	378 (96.68)	204 (100)
Educational Status;		
Primary School	5 (1.28)	0 (0)
Secondary School	105 (26.85)	60 (29.41)
Tertiary	281 (71.87)	144 (70.59)
Parity;		
0	119 (30.43)	50 (24.51)
1–4	267 (68.29)	149 (73.04)
≥ 5	5 (1.28)	5 (2.45)

In all, 45 of the participants had GDM while 8 had Diabetes in Pregnancy (DIP), giving a point prevalence of 11.51% and 2.05 for GDM and DIP respectively, in the universal group. Thus, 53 participants (13.55%; 53/391) had hyperglycemia in pregnancy (GDM plus DIM). Using selective risk factor based screening, 31 (7.93%; 31/391) of the participants had GDM while 8 (2.05%, 8/391) had DIP. Giving a selective risk factor based screening miss rate of 31.11% (14/45) for GDM. However, all the 8 participants with DIP in this study were picked by the selective risk factor based screening.

One hundred and eighty seven (47.83%) of the participants had no risk factors among which 14 (7.49%) had GDM. The sensitivity, specificity, positive predictive value and negative predictive value were 73.58, 48.82, 19.12 and 92.51% respectively for selective screening modality as showed in Table 2.

**Table 2:** Performance of Risk factor based screening for Hyperglycemia

	≥1Risk Factor	No Risk Factor	Total
Hyperglycemia	39 (9.97%)	14 (3.58%)	**53 (13.55%)**
No Hyperglycemia	165 (42.20%)	173 (44.25%)	**338(86.45%)**
Total	**204(52.17%)**	**187(47.83%)**	**391(100%)**

Sensitivity = 73.58%; Specificity = 48.82%; Positive Predictive Value = 19.12%; Negative Predictive Value = 92.51%

**Fig. 1:**
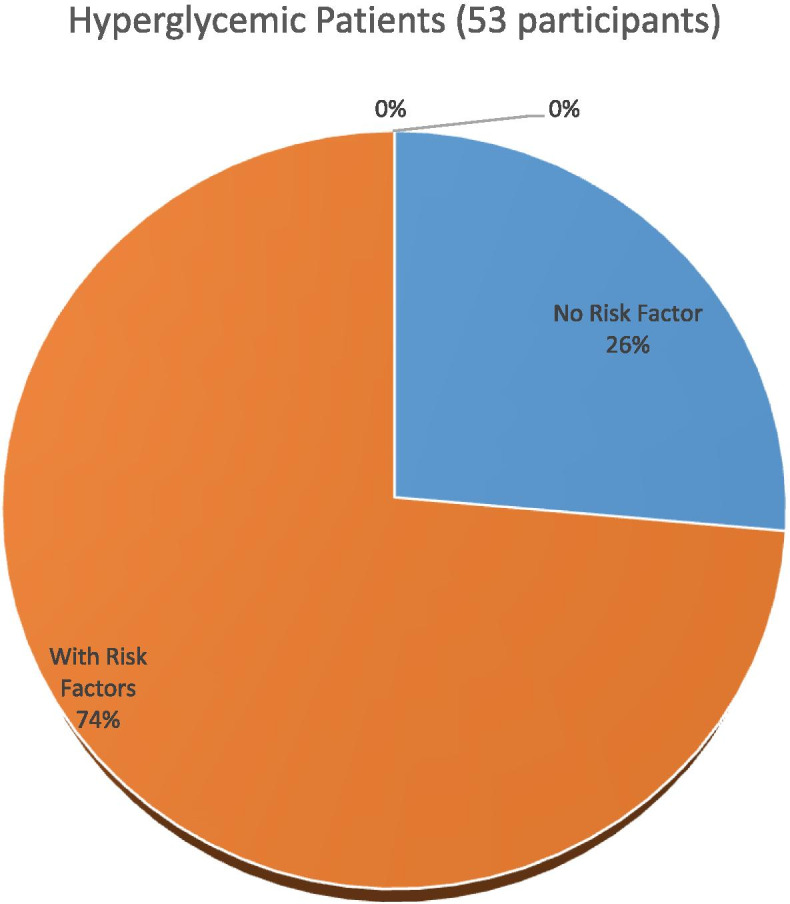
Hyperglycemic patients with versus without risk factors

Figure 1 is a Pie chart showing total number of patients with hyperglycemia with and without risk factors, 39 (74%) versus 14 (26%) respectively.

Most (41/53, 77.36%) of those with hyperglycemia were positive for the fasting blood sugar out of which 34 (64.15%) were in the selective group. Nine (2.30%) of those with hyperglycemia were positive for all the three tests in the universal group and were all in the selective group as showed in Table 3.

**Table 3:** Number of women diagnosed with Fasting Blood Sugar, 1 h post glucose load, 2 h post glucose load and all three respectively

Test	Universal n = 391 (100%)	Selective n = 204 (100%)
Fasting Blood Sugar:		
Gestational Diabetes Mellitus	41 (10.49%)	34 (16.67%)
Normal	350 (89.51%)	170 (83.33%)
One-Hour Post Glucose Load:		
Gestational Diabetes Mellitus	27 (6.91%)	18 (8.82%)
Normal	364 (93.09%)	186 (91.18%)
Two-Hours Post Glucose Load:		
Gestational Diabetes Mellitus	20 (5.12%)	16 (7.84%)
Normal	371 (94.88%)	188 (92.16%)
All Three Tests:		
Gestational Diabetes Mellitus	9 (2.30%)	9 (4.41%)
Normal	381 (97.70%)	195 (95.59%)

Table 4 compared the mean of some numerical variables of age, weight, mid arm circumference, systolic and diastolic blood pressures of those with hyperglycemia and those without. The values were significantly higher in those with hyperglycemia.

**Table 4:** Mean of Numerical Variables of those with compared with those without Hyperglycemia

Numerical Variables	Hypergly Mean ± SD	No Hypergly Mean ± SD	T	*P* Value
Age (years)	32.62 ± 4.76	30.38 ± 5.25	2.89	0.004
Weight (kg)	90.81 ± 15.24	79.81 ± 14.30	5.10	< 0.001
Mid Arm Circumference (cm)	31.42 ± 4.17	29.29 ± 4.32	3.33	< 0.001
Systolic Blood Pressure (mmHg)	112.69 ± 9.52	107.31 ± 12.55	2.96	0.003
Diastolic Blood Pressure (mmHg)	71.15 ± 7.83	66.68 ± 8.31	3.63	< 0.001
Fasting Blood Sugar (mg/dl)	108.65 ± 34.56	71.07 ± 8.79	16.85	< 0.001
1 Hour Post Glucose Load (mg/dl)	178.54 ± 47.22	114.39 ± 18.87	17.55	< 0.001
2 Hours Post Glucose Load (mg/dl)	138.27 ± 38.30	96.04 ± 15.86	13.98	< 0.001

# *Hypergly* Hyperglycemia

On univariate analysis maternal age ≥ 35 years (*p* < 0.001), previous delivery (ies) of macrosomic baby (ies) (p < 0.001), previous unexplained stillbirth(s) (*p* = 0.0012), being a known hypertensive patient (*p* = 0.0125), pre pregnancy body mass index (BMI) of 30 kg/m^2^ or more (*p* = 0.0205), pregnancy weight of 90 kg or more (p < 0.001) and history of previous GDM (p < 0.001) were significantly related to the development of hyperglycemia.

On multivariate analysis, only age of 35 years or more (*p* = 0.0302), pregnancy weight of ≥90 kg (*p* = 0.0117), history of previous GDM (*p* = 0.0415) and being a known hypertensive patient (*p* = 0.0097) were significantly related to the development of hyperglycemia while others were not. However, only 176 (45.01%) of the 391 participants could recall their pre pregnancy weight to enable calculation of their pre pregnancy BMI as showed in Table 5.

**Table 5:** logistic regression for predictors of Hyperglycemia

Predictors	hypergly	No Hypergly	Uni Analysis. OR; (95% Ci); *P* Value	Multi Analysis. OR; (95% Ci) P Value
Age ≥ 35 years	19	47	3.9674;(2.0933–7.5192); ***p*** **< 0.001**	6.6425;(1.5745–77.0852);***p*** **= 0.0302**
Previous Macrosomic Baby	20	56	3.1362;(1.6736–5.8767); **p < 0.001**	2.4565;(0.5385–11.2061); ***p*** **= 0.2458**
Previous Unexplained Stillbirth	6	2	35.7447;(4.0869–312.6306); **p = 0.0012**	3.3703;(0.2083–54.5221); ***p*** **= 0.3923**
History Of Previous GDM	6	2	21.9130;(4.2947–111.8077); **p < 0.001**	70.1720;(1.1780–4180.0549); **p = 0.0415**
Known Hypertensive Patient	5	9	4.3883;(1.3779–13.9757); ***p*** **= 0.0123**	42.4353;(2.4776–726.8075); **p = 0.0097**
Pre-Pregnancy BMI ≥ 30 kg/m^2^	17	39	2.5164;(1.1525–5.4946); **p = 0.0205**	1.0005;(0.2712–3.6911); ***p*** **= 0.9994**
Weight In Index Pregnancy ≥90 kg	37	75	8.6498;(4.5049–16.6084)**p = < 0.001**	7.5706;(1.5679–36.5533); **p = 0.0117**
First Degree Relative With DM	5	60	0.4911;(0.1874–1.2869); ***p*** **= 0.1480**	0.5168;(0.1403–1.9042); ***p*** **= 0.5959**

# *Hypergly* Hyperglycemia, *Uni* Univariate, *Multi* Multivariate, *OR* Odd Ratio

Among the participants 5.63% (22/391) experienced some side effects in the course of the Oral Glucose Tolerance Test, which included vomiting (1.28%, 5/391), nausea (2.05%, 8/391) and abdominal discomfort (2.30%, 9/391) as shown in Table 6. These were mild and transient.

**Table 6:** Side effects of Glucose solution observed among the participants

Side Effect	Number n = 391 (100%)
Vomiting	5 (1.28%)
Nausea	8 (2.05%)
Abdominal Discomfort	9 (2.30%)

## Discussion

The point prevalence of GDM in this study using universal screening and adopting the international Association of Diabetes in Pregnancy Study Group (IADPSG) diagnostic criteria[27] was 11.51 and 7.93% for selective screening. This shows that the burden of GDM in our antenatal clients is high both for universal and selective modalities but significantly higher for the universal approach. This figure is more than the 3.30 and 8.30% prevalence reported in Uyo, Akwa-ibom State and Jos Plateau state respectively. In these studies universal screening modality were equally adopted but the 1999 WHO criteria, with higher fasting blood glucose cut off, was used in making the diagnosis[5, 10]. Thus, the lower prevalence in those studies. Diet rich in fish is believed to reduce GDM risk and may partly explain the lower prevalence among the riverine Uyo population while predominantly starchy diet increase the risk of GDM [31] as may obtain in this study population in Abakaliki, Ebonyi State where carbohydrate is the staple food. The prevalence of GDM in this study is however lower than 19 and 25.8% prevalence of GDM reported in Owerri Southeast Nigeria [30] and South African antenatal population [16] respectively using similar screening and diagnostic modalities as were used in this study. The difference may not be unrelated to the sample size of the study and the prevalent lifestyle in the study population. While the sample size for the study done in Owerri was only 100 participants[30], the study done in South Africa reported lifestyle changes that led to increased obesity in their study population[16].

Evaluation of pregnant women for GDM based on risk factors alone (selective screening) has been shown to be associated with significant number of missed cases of GDM[25]. The selective screening miss rate for GDM in this study was 31.11% (14/45), which is high. This is of particular importance since those who have GDM without risk factors are still at increased risk of developing both the fetal/neonatal and maternal complications of GDM[24]. Also, it has been shown that diagnosis and treatment of cases of GDM reduces the risk of fetal/neonatal and maternal complications[2] thus the need to properly diagnose and treat all cases of GDM. The risk factor based miss rate in this study was less than 33–50.7% reported in other studies [24] but higher than 23.8% miss rate reported in Malaysia [32]. This could be occasioned by differences in diagnostic modality/criteria used in the different studies and racial differences in the study populations.

Selective screening for GDM has been shown to have poor sensitivity and poor positive predictive value[16, 24]. The sensitivity, specificity, Positive Predictive Value and Negative Predictive Value for selective screening in this study were 73.6, 48.8, 19.1 and 92.5% respectively. These are low but for the negative predictive value. Thus, are not optimal for screening and diagnosis of GDM which has known preventable[4, 6, 12] fetal/neonatal and maternal complications[2, 20–28, 30]. The result from this study is in keeping with the findings in others studies[16, 25]. The universal screening is therefore recommended because selective screening leads to missing of about one third of the women with GDM[16, 25].

In this study the risk factors that had statistically significance association with the development of hyperglycemia on univariate analysis were maternal age ≥ 35 years, previous macrosomic baby (ies), previous GDM, previous unexplained stillbirth, pre-pregnancy body mass index (BMI) ≥30 kg/m^2^, being a known hypertensive patient and pregnancy weight ≥ 90 kg. These are in keeping with findings in other studies[5, 10, 16, 19, 20] though in this study having first degree relative(s) with diabetes mellitus was not significantly related to the development of hyperglycemia. The presence of first degree relative(s) with diabetes mellitus was equally not significant in a similar study[10]..The differences in the predictors of hyperglycemia in different studies shows that all the significant predictors of hyperglycemia may not have been identified and there may be predictor variability among different study populations.

However, on multivariate analysis, only age of ≥35 years, pregnancy weight of ≥90 kg, history of previous GDM and being a known hypertensive patient were significantly related to the development of hyperglycemia while others were not. Older maternal age, high pregnancy weight and high blood pressure as well as pre-pregnancy overweight were significantly related to the development of hyperglycemia on multivariate analysis in other studies[5, 27]. Previous GDM[5, 27], and previous macrosomia[27] did not predict the development of hyperglycemia in some other studies. High pre-pregnancy BMI was not significant on multivariate analysis in this study, even though pre-pregnancy obesity is a known strong independent predictor of GDM [32]. This is probably because only 45% (176/391) of the study participants remembered their pre-pregnancy weight, which may not be representative of the whole study population. This introduced recall bias which is a known problem of selective screening for hyperlycemia[20]. In Jos[10] north central Nigeria, previous history of fetal macrosomia was the only factor noted to be significantly related to the development hyperglycemia on multivariate analysis.

The observed side effects of the glucose solution were few, mild and transient as were noted in another study [32]. This shows that the 2 h hour 75 g OGTT is generally tolerable and safe in pregnancy and can be used to assess for GDM without significant concerns for side effects.

## Conclusion

Universal screening is superior to selective risk factor based screening for GDM as it was able to identify 31.11% of patients missed on risk based screening. The predictors of GDM in this study are increased maternal age, obesity, history of previous GDM and Hypertension.

Further studies are however needed to explore if women diagnosed with GDM without risk factors have the same risks of adverse pregnancy outcomes as those with risk factors.

## Recommendation

Universal screening using one step 75 g OGTT, which is diagnostic for GDM when abnormal, is recommended for our antenatal clients because it has higher pickup rate and it is safe.

## Data Availability

The datasets used and/or analysed during the current study available from the corresponding author on reasonable request.
